# Selected Skill Sets as Building Blocks for High School-to-Medical School Bridge: Longitudinal Study Among Undergraduate Medical Students

**DOI:** 10.2196/43231

**Published:** 2023-07-04

**Authors:** Laila Alsuwaidi, Farah Otaki, Amar Hassan Khamis, Reem AlGurg, Ritu Lakhtakia

**Affiliations:** 1 College of Medicine Mohammed Bin Rashid University of Medicine and Health Sciences Dubai United Arab Emirates; 2 Strategy and Institutional Excellence Mohammed Bin Rashid University of Medicine and Health Sciences Dubai United Arab Emirates; 3 Hamdan Bin Mohammed College of Dental Medicine Mohammed Bin Rashid University of Medicine and Health Sciences Dubai United Arab Emirates

**Keywords:** transition, undergraduate, medical, education, academic performance, self-regulated learning

## Abstract

**Background:**

The high school–to–medical school education transition is a significant milestone in the students’ academic journey, which is characterized by multiple stressors. Although this crucial transition has been repetitively explored, the concept of proactively intervening to support this transition is still novel.

**Objective:**

In this study, we investigated the efficacy of a web-based multidimensional resilience building intervention in developing selected soft skills that are believed to drive the learner’s success in any learning setting. The association between the students' academic performance over time and their proficiency in selected modules addressing skill sets, including Time Management, Memory and Study, Listening and Taking Notes, and College Transition, was also assessed to test the impact of the intervention on the students’ learning.

**Methods:**

A longitudinal study was conducted on 1 cohort of students of a Bachelor of Medicine, Bachelor of Surgery program (MBBS). The medical students were offered a learning intervention around 4 skill sets during the first year of the 6-year program. Quantitative analyses were conducted using deidentified data, relating to the students' proficiency in the 4 skill sets and to the students’ academic performance: grade point average (GPA). Descriptive analyses constituted computing an overall score of skill sets’ proficiency (of all 4 selected skill sets). The mean and SD (and percentage of the mean) were also calculated for each skill set component, independently, and for the overall score of skill sets’ proficiency. Bivariate Pearson correlations were used to assess the extent to which the academic performance of the students can be explained by the corresponding students’ level of proficiency in each skill set component and by all 4 sets together.

**Results:**

Out of the 63 admitted students, 28 participated in the offered intervention. The means and SDs of the annual GPA of the students for years 1 and 2 (GPA range 1-4) were 2.83 (SD 0.74) and 2.83 (SD 0.99), respectively. The mean and SD of the cumulative GPA toward the end of year 2 was 2.92 (SD 0.70). Correlation analysis showed that the overall score of skill sets proficiency was significantly associated with the annual GPA of year 1 (*r*=0.44; *P*=.02) but was not associated with their annual GPA of year 2. The cumulative GPA (toward the end of year 2) appeared to be significantly associated with the overall score (*r*=0.438; *P*=.02).

**Conclusions:**

Developing purposefully selected skill sets among medical students holds the potential of facilitating the high school–to–medical school education transition and is likely to improve their academic performance. As the medical student progresses, the acquired skills need to be continuously reinforced and effectively built upon.

## Introduction

High school–to–medical school education transition is a significant milestone in the students’ academic journey. The transition entails a physical and mental multidimensional adaptation to higher education frameworks and their expectations, self-regulated behaviors, and sociocultural and environmental influences [1]. A teacher-driven structured, planned, monitored, and evaluated school program leaves the school-leaver unprepared for becoming an independent autonomous sophomore, and for inhabiting an open campus with a potentially experimental lifestyle [2]. This highlights the importance of self-regulated learning (SRL), and of crafting nurturing environments that inspire and empower students to create their own learning pathways. SRL relates to 5 elements of the individual students: cognitive and metacognitive, behavioral, and motivational and emotional [3]. Self-regulated students are recognized as active learners, managing their own learning via monitoring and the use of metacognitive strategies [4]. Multiple transition points in health professions’ education, first at admission to medical school, second from preclinical to clinical years of learning, and finally from clinical years to practice, demand adaptation by the students and nurturing by the educators and by other environmental support mechanisms [5-10].

The students’ perception of their capabilities of coping with their workload affects their ability to achieve their academic goals [11,12]. A framework for comprehensive and coherent development of learning proposes a preinduction web-based course followed by a carefully designed induction phase with increasing personal tutor support and constant self-reflection by the student [13,14].

The difficulties students confront are variably coped with depending upon the entry level of a medical program (ie, undergraduate or graduate) and on individual-level characteristics. In all cases, unfavorable impacts can range from suboptimal academic performance to adverse health outcomes, requiring attempts at prevention, early detection, and mitigation [15]. Although this crucial phase of the educational transformation is both documented and has earned scientific exploration, programs that bridge and support the high school–to–medical school education leap are a recent phenomenon [14]. Outcomes of such interventions have also not been extensively published, discussed, or translated into policy [2]. A “learning to learn” framework supported moving away from the deficiency model of focusing on remedying missing skills during the high school–to–medical school education transition [2]. Instead, a “holistic subject-specific approach” that supports the engagement and commitment of academic teachers to ensure the growth of independent learners was proposed.

This study was therefore undertaken to implement and analyze the impact of a web-based multidimensional resilience building foundational program, designed to foster students’ SRL. This intervention ran synchronously during the first curricular year of an undergraduate Bachelor of Medicine, Bachelor of Surgery program (MBBS). Through this study, we investigated the efficacy of this intervention. We also analyzed the association between the students' proficiency of 4 purposefully selected sets of skills and their academic performance over time. Accordingly, our research question was: is the proficiency in the selected skill sets associated with the students’ academic performance?

## Methods

### Ethical Considerations

The ethics approval for this study was granted by the Mohammed Bin Rashid University of Medicine and Health Sciences-Institutional Review Board (MBRU-IRB-2021-58). Informed consent was obtained from all the participants. All methods were performed in accordance with relevant guidelines and regulations. Consent for publication was not applicable as there are no individual details, images, or videos.

### Context of the Study

The study was conducted at the College of Medicine at the Mohammed Bin Rashid University of Medicine and Health Sciences (MBRU) in Dubai, United Arab Emirates, on a cohort of undergraduate medical students. Students are admitted into the MBRU 6-year MBBS directly from high school with no premedical foundation year. The MBBS is divided into 3 phases, each of which has several components (phase 1: 1 year; phase 2: 2 years; and phase 3: 3 years). Student progression to the next phase is subject to successful completion of the progression requirements along with the achievement of a minimum cumulative grade point average (cGPA) at the end of the preceding phase.

### Description of the Intervention and Study Participants

In the academic year 2018-2019, a total of 63 students (52 females and 11 males) were admitted to the MBBS at MBRU. The cohort intake was homogenous with respect to age and academic credentials, given the standardized admission selection processes and procedures to test cognitive and noncognitive abilities. To ease the transition of the high school students admitted to the medical school, and to enable personal, academic, and professional development, the students were offered a web-based multidimensional resilience building intervention, which is a proprietary commercial program (Pearson College and Career Readiness Solution-2018) [16].

**Figure 1:**
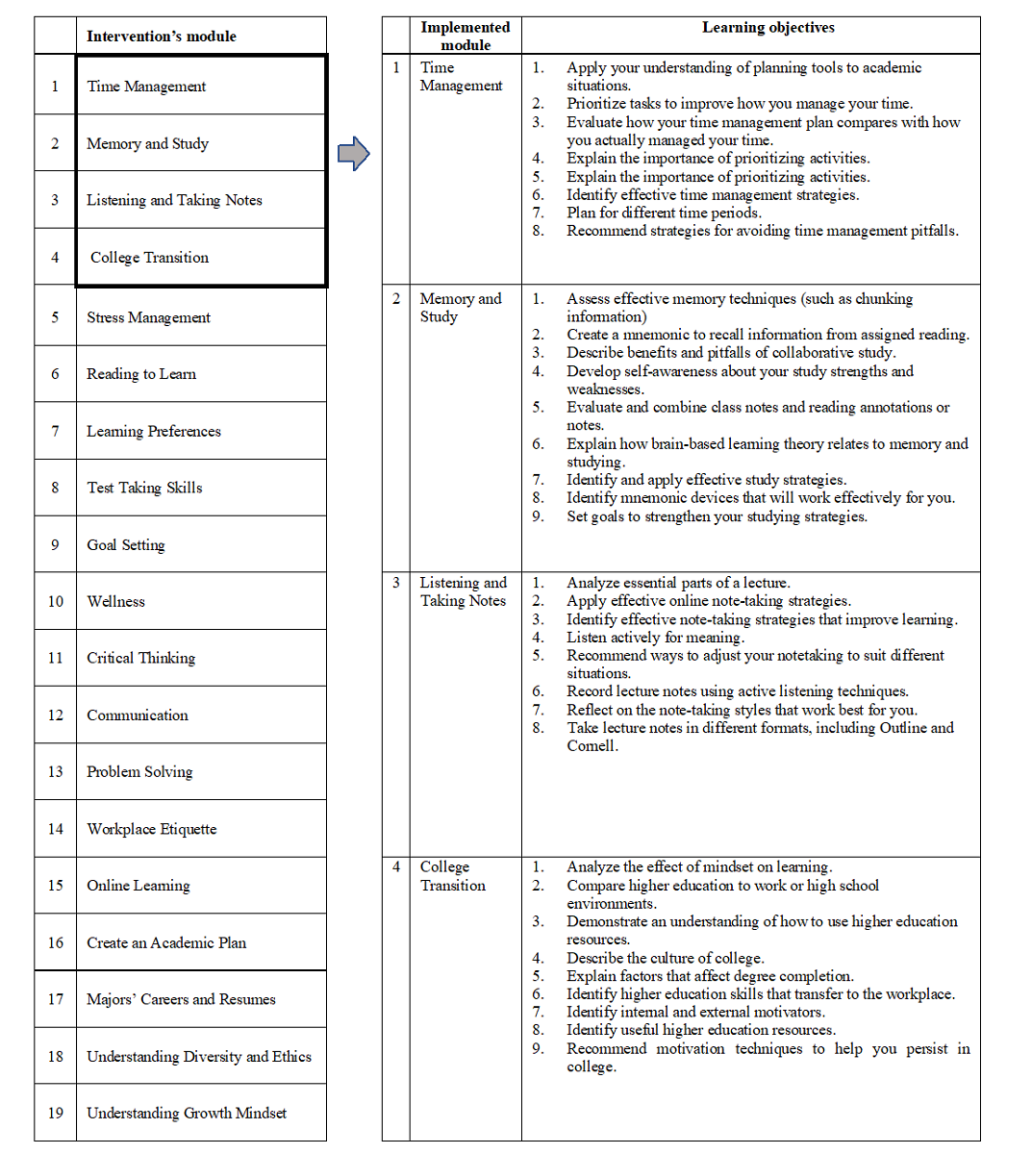
The intervention’s modules and corresponding learning objectives of the implemented modules.

The adapted intervention was developed by the system provider in alignment with the personal and social capabilities framework. This framework pinpoints crucial sets of soft skills that are believed to increase the users’ awareness, happiness, empathy, and resilience. All of which are necessary for a successful high school–to–medical school transition [17]. The tool’s developer identified 6 common categories of soft skills that can drive the learner’s success in any learning setting: (1) collaboration and teamwork; (2) communication; (3) critical and creative thinking; (4) leadership; (5) self-management/initiative and mindset; and (6) social responsibility [18]. The proposed digital tool consists of 19 web-based modules listed in Figure 1. Multimedia Appendix 1 provides a detailed description of the tool.

The implementation of the intervention was spearheaded by an Advising Group composed of a selection of students’ academic advisors from the MBBS (all of whom are faculty members), in addition to professional administrative and technical members from the MBRU. The intervention was implemented in alignment with the SRL theories. Accordingly, the adaption occurred in 3 phases that are common across the main theories of SRL: preparatory, performance, and appraisal [19,20].

Each student was given a unique number and access code to the web-based tool on the orientation day at the start of the respective academic year. The use of the tool was not mandated. The students were offered an information session conducted during the orientation day to introduce the tool and to address the students’ queries. The participants’ first exposure was during the new students’ orientation at the beginning of the academic year, where representatives of the advising group facilitated the students in the initiation of the preparatory phase. Thereafter, the students were enabled to deploy the 4 components integral to SRL: task definition, goal setting and planning, enacting study tactics and strategies, and metacognitively adapting studying [4]. Throughout the performance phase, the academic members of the advising group played the role of mentors, where they assumed that the students are self-directed, intrinsically motivated, have previous knowledge and experience, will form mental models through this learning and development experience, and use analogical reasoning as their knowledge base evolves [21]. Thus, throughout the assigned 6 weeks, 3 reflection sessions (1 every 2 weeks) were conducted. This was done to foster SRL in the context of collaborative learning [22]. Four out of the 19 modules of the adapted web-based program were prioritized and purposefully selected as part of this transition. The selection was based on the perceived deficiencies identified at the same learning stage in previous intakes of the respective MBBS. Accordingly, the selected modules addressed the following skill sets: Time Management (TM), Memory and Study (MS), Listening and Taking Notes (LTN), and College Transition (CT). The tool is supplemented with pre- and postassessments, and each module has learning objectives where the attainment of the corresponding objectives is gauged by the post-assessment. The outcome of the assessment is reflected on a “Mastery Report” generated for individual students as the *appraisal phase.*

### Data Collection

Data related to the students' proficiency in the 4 selected sets of skills were extracted from the reporting dashboard (from the corresponding “Mastery Report”). This is linked with grade book, embedded in the software of the web-based tool and corresponds to the learning outcomes of the courses (Figure 1). Each set of skills corresponds to a module, with a list of preset learning objectives. Attainment of a learning objective is represented with “1” (versus “0”). The number of learning objectives varies from one module to another. For the set of skills under investigation, the number of learning objectives was as follows: 8 for TM, 9 for MS, 8 for LTN, and 9 for CT.

As for the data related to the students’ academic performance, the cumulative or semester grade point average (GPA) for the students was retrieved from the student information self-service. The extracted information from the students’ records was retained in such a manner that subjects cannot be identified. Data were coded and linked through identifiers to the subjects.

### Data Analysis

The quantitative data were analyzed using SPSS for Windows (version 27.0; IBM Corp).

The descriptive analysis constituted computing an overall score of skill sets’ proficiency (of all 4 selected skill sets). The highest possible score is 34 (ie, the sum of the learning objectives, where attaining the respective objective corresponds to “1” and failing to attain it corresponds to “0”) and the least possible is 0. Then, the mean and SD (and percentage of the mean) were calculated for each skill set component, independently, and for the overall score of skill sets’ proficiency. The validity tests of Cronbach α and the principal component analysis (PCA) of the Kaiser-Meyer-Olkin and Bartlett’s test were performed to ensure the internal consistency and check the external variance, respectively, of the overall score of skill sets proficiency, and that of each skill set component independently (since each component is comprised of a set of skills; Figure 1).

To select the appropriate comparative analysis tests, a test of normality (Kolmogorov-Smirnov) was conducted for the annual GPA of years 1 and 2 and of cGPA. The data of the annual GPA of year 1 and that of the cGPA turned out to be normally distributed (*P*=.11 and *P*=.15, respectively). As for the data of the annual GPA of year 2, it turned out to be nonnormally distributed (*P*=.005). Given the fair sample size, the bivariate Pearson correlations were used to assess the extent to which the academic performance of the students (GPA1, GPA2, and cGPA) can be explained by the corresponding students’ level of proficiency of each skill set component and by all 4 sets together (ie, the overall score of skill sets’ proficiency).

## Results

Out of the 63 admitted students, 28 (27 females and 1 male; 44.44%) participated in the above-mentioned intervention (ie, 44.44%). Table 1 presents the participants' demographic details.

**Table 1:** Participants' demographic details (N=28).

Items		Values, n (%)
**Sex**		
	Male	1 (4)
	Female	27 (96)
	Intersex	0 (0)
**Nationality**		
	UAE^a^	12 (43)
	Non-UAE	16 (57)
**High school classification**		
	Private	28 (100)
	Government	0 (0)
**Curriculum**		
	American	17 (60)
	British	5 (18)
	Indian	3 (11)
	International Baccalaureate	3 (11)

^a^UAE: United Arab Emirates.

The reliability score of Cronbach α for the overall score of skill sets’ proficiency was 67%. When each skill set component, TM, MS, LT, and CT was analyzed independently, and Cronbach α scores were 93%, 88%, 69%, and 81%, respectively. The percentage of the total average of the overall score of skill sets’ proficiency turned out to be 32.15%, as per Table 2. According to the PCA (Kaiser-Meyer-Olkin Measure of Sampling Adequacy), most of the variance can be explained by the instruments of each skill set component and the overall score of skill sets proficiency, which means this instrument is not only reliable but also, according to Bartlett’s test of Sphericity, valid to measure what it is intended to measure (*P*<.001).

**Table 2:** The percentage of the mean and SD for each skill set component and for the overall score of skill sets’ proficiency.

Module	Items (ie, highest possible score), n	Mean (SD)	Percentage of the mean
TM^a^	8	2.46 (2.82)	30.75
MS^b^	9	3.36 (2.87)	37.33
LTN^c^	8	2.39 (1.93)	29.88
CT^d^	9	2.71 (2.42)	30.11
Overall	34	10.93 (7.20)	32.15

^a^TM: Time Management.

^b^MS: Memory and Study,

^c^LTN: Listening and Taking Notes.

^d^CT: College Transition.

The mean and SDs of the annual performance of the students for years 1 and 2 on a GPA range of 1-4 were 2.83 (SD 0.74) and 2.83 (SD 0.99), respectively. The mean and SD of the cGPA at the end of year 2 was 2.92 (SD 0.70). In Table 3, the bivariate Pearson correlations showed that the overall score of skill sets proficiency was significantly associated with annual academic performance of the students in year 1 (*r*=0.44; *P*=.02) but was not associated with their annual academic performance in year 2 (*r*=0.327; *P*=.09). Yet, the cGPA (toward the end of year 2) appeared to be significantly associated with the overall score (*r*=0.438; *P*=.02). Also, the performance of the students seemed not to be associated with their proficiency scores in each of the components, independently.

**Table 3:** The output of the bivariate Pearson correlations.

Characteristics		Overall score
**Annual GPA^a^-Y^b^1**		
	Correlation	0.440
	Significance	.019^c^
	Sample, N	28
**Annual GPA-Y2**		
	Correlation	0.327
	Significance	.089
	Sample, N	28
**cGPA^d^-Y1 and Y2**		
	Correlation	0.438
	Significance	.020^c^
	Sample, N	28

^a^CPA: grade point average.

^b^Y: year.

^c^Correlation is significant at the .05 level (2-tailed).

^d^cGPA: cumulative grade point average.

## Discussion

### Principal Findings and Comparison With Prior Work

The start of the educational journey in medical schools requires building of resilience through early mastery of time management, modification of study methods to cope with quantum of cognitive burden, and evolution toward higher levels of analytical thinking [6,7]. It entails the recognition of the need for self-reliance and peer collaboration, reorientation to resources, and development of the capacity to handle success and reverses. The medical novice with little prior exposure to disease and death requires a framework of resilience within which they develop a new professional identity [8,9]. Tests of knowledge, skills, and competencies in a medical curriculum require both proficiency and test understanding, which are key to success and contribute to perceived self-worth [10].

In a scoping review of learning support intervention programs, during the first year of medical school, it was found that interventions could be identified as proactive or reactive addressing deficits or promoting development [14]. The interventions addressed knowledge, personal and professional learning skills, and program learning elements and were delivered through a variety of institutional stakeholders and student-centered initiatives. This study showed that the intervention of developing the following skill sets, TM, MS, LTN, and CT, constituted an efficacious bridge in terms of facilitating high school-to-medical school transition. The proficiency of the students in the respective skill sets, altogether, was significantly associated with enhanced performance in the first year (ie, annual GPA-Y1) and cumulatively toward completion of the second year of the MBBS (ie, cGPA-Y1 and Y2). The results show that entry to an undergraduate medical program entails a transition that calls for students’ adaptation to the medical curriculum and a process of professional identity building [8,23]. In fact, this study established that developing the combination of all 4 selected skill sets is what adds value (together and not in isolation) toward adapting to this transition, as reflected in the participants’ academic performance. Therefore, the web-based intervention under investigation offered a holistic, multipronged solution to a compounded transition challenge, where it was evident that the integrated whole, in terms of the educational offerings, was more than the sum of its individual components.

The intervention, investigated in this study, appeared efficacious in the first year (ie, annual GPA-Y1) but not in the second year (ie, annual GPA-Y2). In other words, the proficiency in the selected skill sets successfully predicted academic performance in the first year. However, as the students progressed to the second year, the true, intended effect of the intervention appeared to have dissipated. This finding supports the provision of a refreshing course to reinforce the benefits initially accrued. It would also be helpful to offer the students complementary learning opportunities of more advanced and focused skill sets, appropriate to their next stage of learning. Of note, study time and study habits are known to have a variable relationship to performance [6]. In a previously conducted study, student performance in medical school appeared to be better correlated with learning *approaches* rather than learning *styles* [24]. Thus, focusing on adaptive techniques that encourage strategic and deep learning approaches is likely to be most effective in supporting students as they progress in their educational trajectory.

This study also showed that having the intervention was better than not having it. Although the intervention was not efficacious in the second year (ie, annual GPA-Y2), adapting this intervention was still considered beneficial for the students given that the cGPA was significantly associated with the overall skill sets’ proficiency. The tools used in the current study, overall and for each skill set (ie, TM, MS, LTN, and CT), independently, all turned out to be internally consistent or reliable and externally valid. In other words, the components of the tools defined by the web-based intervention under investigation (Figure 1) are worth leveraging as a means of evaluating the proficiency of high school graduates in the selected skill sets and their readiness to transition to universities, in general, and medical schools, in specific. This finding reinforces the importance of basing initiatives aimed at high school–to–university transition on SRL theories, which requires fostering the students’ motivation and commitment to learn [25]. It would also add value to consider not only the persons but also their behaviors and environments, as indicated in the triadic analysis of SRL [19].

The efficaciousness of the intervention under investigation encourages medical educators to think of innovative ways to proactively facilitate not only the entry into medical school transition but also the ones that follow. Next, preclinical to clinical transitions bring novel disruptors due to perceived or actual stress of inadequacies or incompetence which demand tackling through nurturing and empowerment [26]. Finally, transition programs to internship, for example, address “professional reflection, consolidation of knowledge, and social, emotional, and ethical growth” beyond the overt curriculum [27]. Transition-to-residency pilot programs have been hailed as acceptable and feasible mechanisms to make the final transition to graduate studies smoother [28]. With this in mind, we propose an adapted framework of transition support that aligns the timing of the transition support and its context in a stepwise and sustainable fashion. In the context of the MBBS, the early transition support could employ tools that boost communication and a self-management or initiative mindset. In the following preclinical years, increasing levels of critical thinking and collaboration are required as enablers for professional growth. Integrated with clinical years and postgraduate training, social responsibility and leadership would determine the development of the persona of the mature health professional along with academic accomplishment and competency in skills. Accordingly, based on the evidence gathered from this study, we propose a stage-appropriate adaptation support system contextualized to a stepwise transition-mitigation approach to supporting student resilience and progression in a medical education degree program.

### Limitations and Future Directions

This study has several limitations. First, the intervention (in alignment with the ethical principle of autonomy) was not mandatory but made optional. Thus, it was entirely up to the students whether, or not, they wanted to sign up for the offered opportunity. Although it would have been ideal to obtain a higher engagement rate, it is apparently not uncommon for a good proportion of any 1 student body not to sign up to optional learning opportunities [29,30]. This might have introduced a bias, where, for example, those who chose to take part in the experience were the ones who were more competent and perhaps better at self-directed learning. Second, the participants constituted a sample of a single cohort (with a low response rate). Hence, the generalizability of this study’s findings is limited. The findings of this study, however, can be transferred to student populations that are characteristically similar to those under investigation. It will be worthwhile to conduct follow-up studies that compare several such programs across multiple institutions, preferably in different countries. Finally, our study did not focus on nonscholastic aspects of the student experience, which could help evaluate noncurricular stressors that either contribute to or aggravate student nonprogression. Such variables could be of relevance given the complementarity of the social, cultural, symbolic, and economic capitals to the student’s capital in determining both the intent to join medical school and the achievement of goal posts (while navigating medical graduation) [30,31]. A study of such parameters would help in designing a 360-degree plan of action that begins before entry into the medical program, molds to the progression level needs, and in later years, provides seamless support to transit to graduate medical education and the health professions workspace.

### Conclusions

This study highlights the importance of developing a contextualized, evidence-driven intervention to proactively nurture purposefully selected skill sets among medical students to facilitate their education transitions, and in turn their academic performance and progression. Such an intervention should not be perceived as a 1-time learning bridge around high school–to–medical school but rather a series of initiatives that address the specific needs of medical students, depending on the stage of their educational trajectory. This cascade of events will build upon each other, continuously reinforcing the acquired knowledge and skills. We recommend for all such activities to focus on empowering medical students and fostering their capacity for SRL.

## Appendix

Multimedia Appendix 1 Intervention tool description.

## Abbreviations

cGPA: cumulative grade point average
CT: College Transition
GPA: grade point average
LTN: Listening and Taking Notes
MBBS: Bachelor of Medicine Bachelor of Surgery program
MBRU: Mohammed Bin Rashid University of Medicine and Health Sciences
MS: Memory and Study
PCA: principal component analysis
SRL: self-regulated learning
TM: Time Management

## Data Availability

The data collected in this study are included in the published manuscript and are available with the corresponding author.
