# Supplementary material for: Selected Skill Sets as Building Blocks for High School-to-Medical School Bridge: Longitudinal Study Among Undergraduate Medical Students
Source: JMIR Med Educ. 2023 Jul 4;9:e43231. doi: 10.2196/43231 (PMC10354651; doi:10.2196/43231)
Supplement: Multimedia Appendix 1 [file mededu_v9i1e43231_app1.pdf]

1. Executive Summary

MBRU strive to give students the knowledge, skills, and strategies to think critically and keep learning — in the classroom and the workplace. MBRU can support your efforts with research-based tools and materials aligned to career readiness standards.

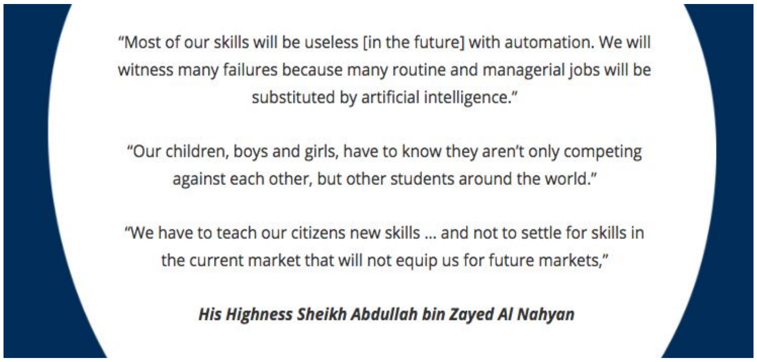

We recognise the great importance of preparing students for employability at MBRU and the special focus in this area meeting the national agenda, and through our unique partnership, Pearson believes that we can support MBRU in delivering an innovative and outcomes-based Career Success progression courses that can benefit all students from highschool to college graduation. The programs will help them set goals and achieve outcomes to progress and succeed throughout their careers.

Together we have a unique opportunity to partner with the common mission to ensure that the top performers in MBRU academically are more equipped with the required skills for the future jobs. This will allow them to feel more confident to conquer the private sector workplace with much more awareness about the soft skills that are required for them to succeed.

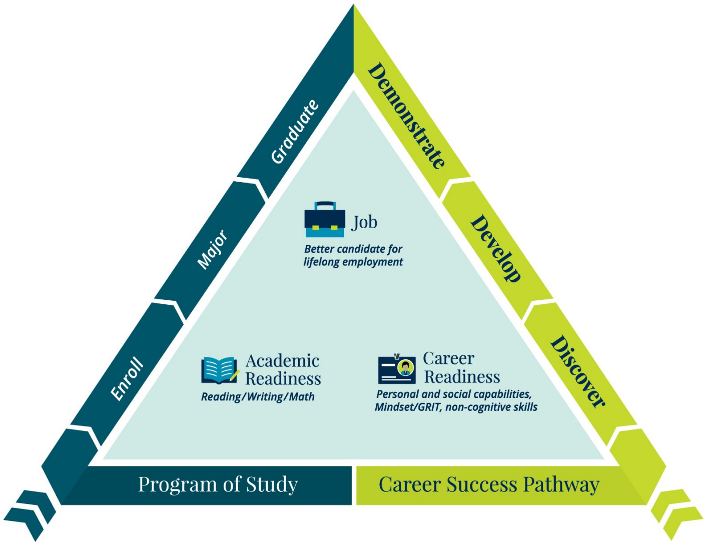

In this proposal we are offering MBRU our newest research outcome based programs- Career Success series to support MBRU at its mission to graduate skilled and top qualified youth in UAE and will be a great indicator and support for the UAE National Agenda Education towards better skilled youth.

To meet today's learner needs, they need to move through BOTH Pathways- Esp the 3 elements of Career Success: Discover, Develop, and Demonstrate. This is where our programs comes in to complement what MBRU is providing it's students to further enhance their soft skills.

Pearson College and Career Readiness Solutions- All rights reserved® 2018

1. College & Career Readiness

We've developed a research based Solution designed to help learners at all college stages develop the personal and social capabilities that are vital for successful 21st century employment. Accordingly to Pearson's approach for students to compete and succeed in both college and career (highlighted in the illustration below), they must be prepared for the challenges they'll face academically and on the job.

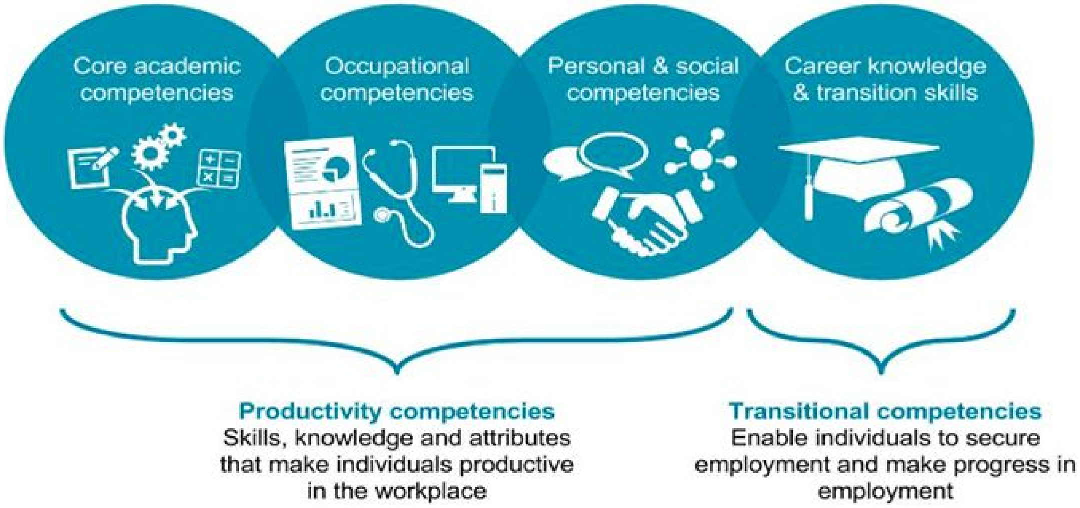

Pearson's Success Solution which is mapped to the Personal and Social Capabilities Framework (PSC)*, effectively empowers ownership, engagement, and accountability, by:

- Connecting a learner's academic plan to his or her career plan
- Replicating the process of choosing a major and practicing academic planning
- Demonstrating student skills through Acclaim badging in GRIT™
- Increasing retention by helping students understand how they spend their time and what adjustments are needed to meet their goals
- Improving the learner's performance
- Revealing a metacognitive data set to correlate with other institution/school data

Pearson College and Career Readiness Solutions- All rights reserved® 2018

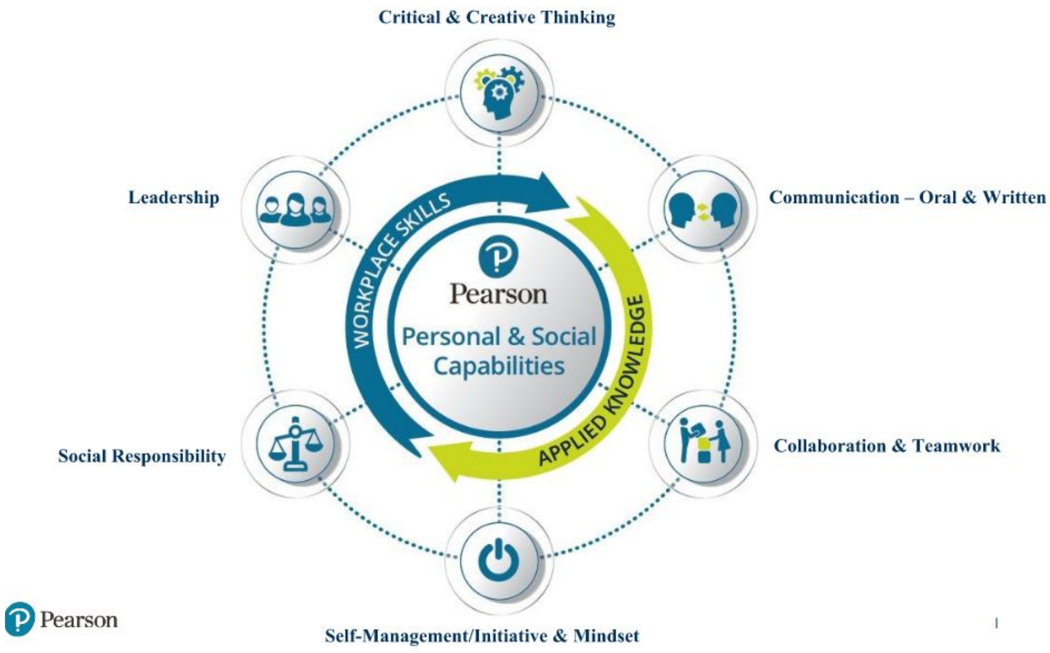

Personal and Social Capabilities Framework:

Personal and social capabilities — also known as soft skills — are critical to employability, a key goal of most learners. Pearson developed a framework to inform the development of educational products and services aimed at bridging the gap between school and work and drive learning outcomes more precisely.

Pearson's PSC Framework was created through an extensive review of existing 21st century skills frameworks, social skills, research related to employability skills, and labor market data. We identified six common categories of soft skills people need to succeed in any employment setting:

- **Collaboration and Teamwork:** Perspective taking, coordination, empathy, trust, flexibility
- **Communication** (Oral & Written):Persuasiveness, presentation skills, active listening, influence
- **Critical & Creative Thinking:** Problem formulation, problem solving, reasoning, extrapolation
- **Leadership:** Managing self, integrity, working with others/teams, managing and planning, vision
- **Self-Management/Initiative & Mindset**:Metacognition, ownership, goal orientation, growth vs. fixed mindset
- **Social Responsibility**: Ethics/integrity, community service, cultural awareness, sustainability

Pearson College and Career Readiness Solutions- All rights reserved® 2018

2. Researchers behind the Program

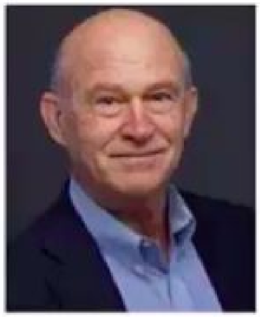

Dr David T. Conley, Ph.D.
Professor, Director, Center for Educational Policy Research, University of Oregon
President, Edimagine Strategy Group
Senior Fellow For Deeper Learning under the sponsorship of Hewlett Foundation

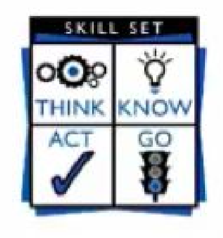

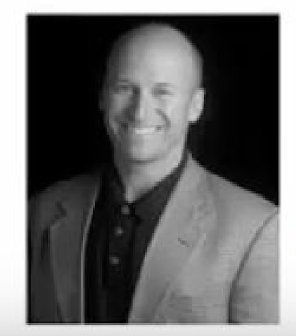

Paul G.Stoltz, Ph.D.
Founder & CEO of PEAK Learning, inc
£1 NY Times Best-selling author of 5 books in 17 languages
Voted “One of the the Top 10 Most Influential Global Thinkers of Our Time” HR Magazine

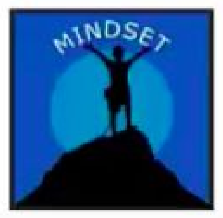

21st Century Learning Strategies

These resources explore why metacognition and the right mindset can make all the difference for learners' success.

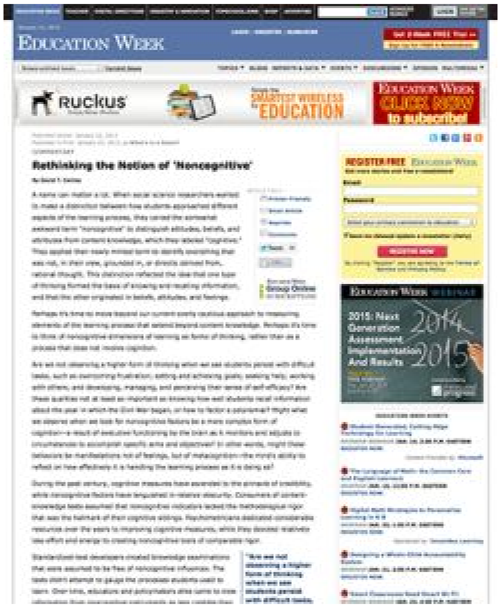

Rethinking the Notion of “Noncognitive”

In this Education Week article, Dr. David T. Conley argues for a closer look at learner skills, attitudes, and techniques, as well as content-knowledge measures, to help all learners succeed.

Pearson College and Career Readiness Solutions- All rights reserved® 2018

3. StudentSuccess Program (Soft Skills)

3.1 CRI Inventory Assessment:

The program is designed to help the learner connect their academic plan to their career plan, through exploration and preparation. The Milestones in the this Learning Path put the process into logical, manageable steps for the learners to explore career options, develop career-relevant skills, and market themselves to potential employers as well as the advancement in their job.

Inventory and assessment for personal and social capabilities or “**soft skills”** by Dr. David T. Conley is a research-based self-diagnostic student inventory that measures/reports college and career readiness around the Four Keys framework (**Think, Know, Act, Go**). It reveals how ready learners are and what it takes to succeed.

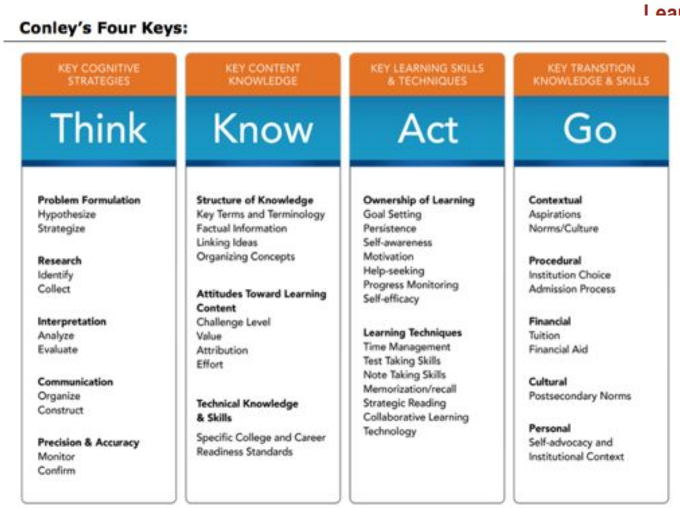

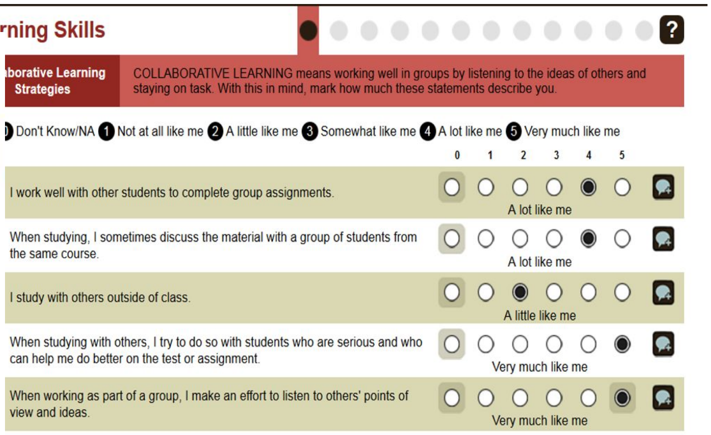

Once learners assess their own readiness, they are able to develop these sought after Personal and Social Capabilities or “soft skills” through activities to apply and transfer what they're learning to their career.

3.2 Outcome of the Student Success Program

Our full Student Success Program will help students build academic and contextual skills. We know that Measurement Matters—and is ongoing in nature. No one is ever an “expert” in “soft skills.” Each topic in the our program is organized by outcomes with peer-led video interviews, interactive practice exercises, and activities to help students master skills and build a foundation for success in academics, career, and life for a holistic profile.

Increase learners’ self knowledge and help them showcase skill.

Pearson College and Career Readiness Solutions- All rights reserved® 2018

4. CareerSuccess Program

4.1 GRIT assessment to assess and grow their GRIT

The GRIT Gauge pre- and post-assessment, by Dr. Paul G. Stoltz, found in the Self-Discovery milestone, is the only validated assessment that measures and reports all facets of GRIT—Growth, Resilience, Instinct, Tenacity, and Robustness. Learners earn a digital credential or badge as evidence of their GRIT mindset. Assignments offer application for this framework and assessment. GRITTY Assignments offer application for this assessment throughout, helping students grow or build their GRIT through journaling, goal setting, and GRIT-specific actions with the ability to earn Acclaim digital credentials in GRIT.

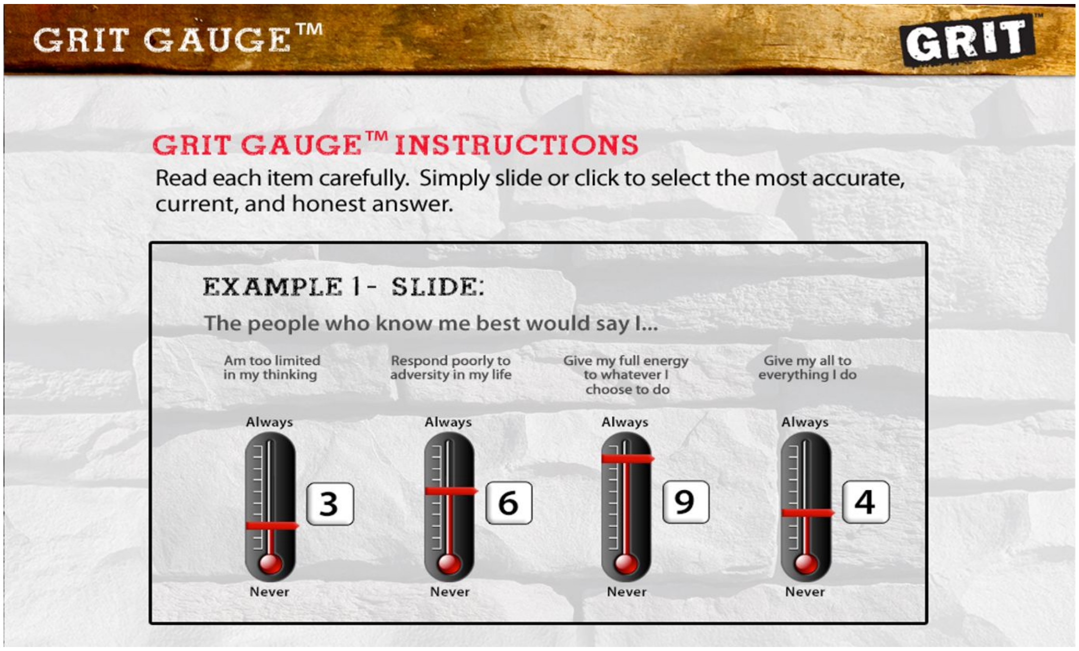

Career Success program which includes GRIT™** and mindset activities that strengthen the learner's ability to reach goals; strategic LinkedIn profile development and networking activities; strategic LinkedIn profile development and networking activities; and support through the embedded coaching sessions.

Pearson College and Career Readiness Solutions- All rights reserved® 2018

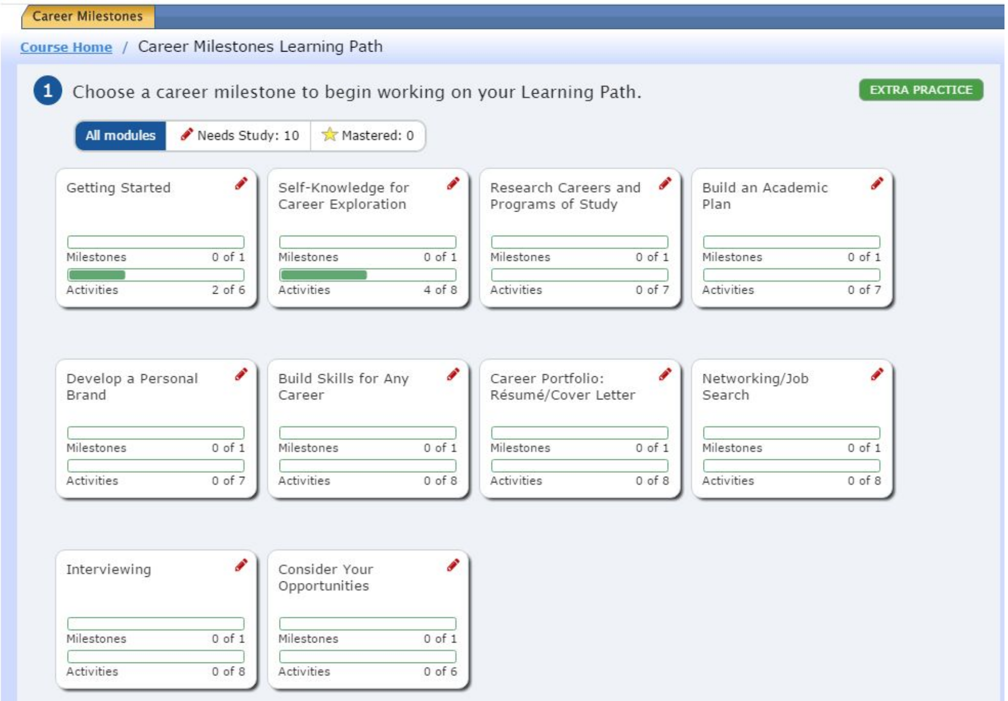

4.2 <u>Outcome of the Career Success Program</u>

Career Success Program helps learners' discover, develop, and demonstrate (the “3 Ds”) their best professional selves to get their targeted job, all while internalizing a process for ongoing personal and professional development that lasts beyond college.

The Nine Milestones in the Learning Path help them discover who they are and how that translates to their academic and career plan, develop transferable skills, and demonstrate their value to potential internships/employers.

Throughout the milestones, they actualize on the 3 Ds through GRIT™ mindset activities and digital credentials that strengthen students’ ability to persist in reaching goals; strategic LinkedIn profile development and networking activities; and career exploration through Labor Market Insight data on jobs.

Upon completion of the program, students will earn Digital badges unlocking the global job economy (shareable on Linkedin)

Once the milestone assets are accomplished and the learner has demonstrated the skills mastery.
Acclaim provides badges from the respected skill whose recognition gets the learners noticed and rewarded. They can easily share their achievements to popular online destinations, and employers can instantly verify their skills.

Pearson College and Career Readiness Solutions- All rights reserved® 2018

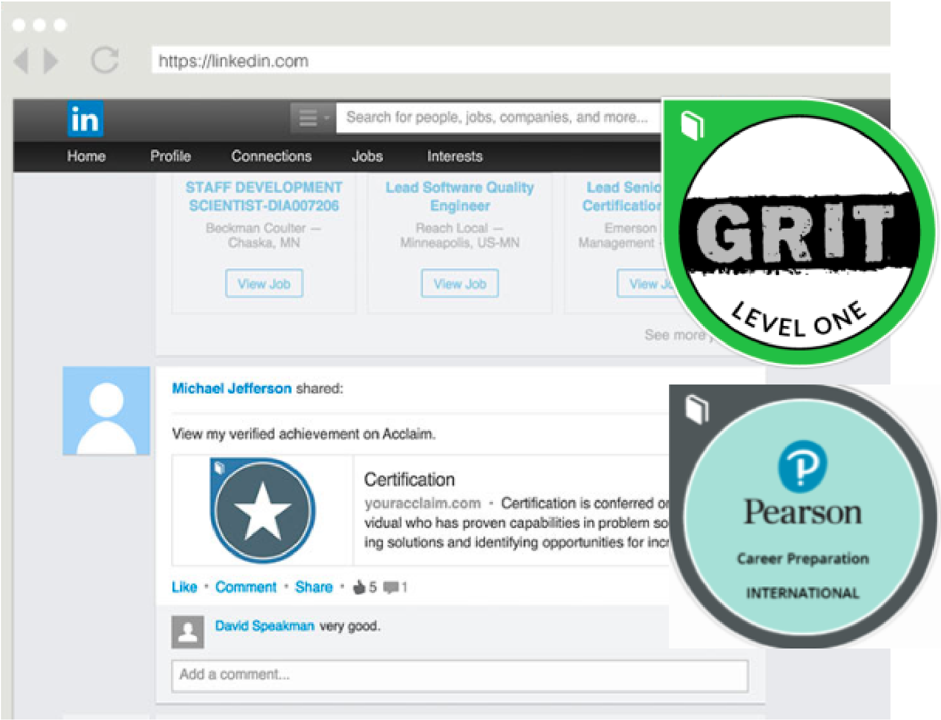

Sharing badges is quick, easy and enhances their professional presence online. They can create a custom message and let everyone know why their accomplishment is important. Anyone who clicks on a shared badge will be able to view all the details on Acclaim for instant verification.

Pearson College and Career Readiness Solutions- All rights reserved® 2018

5. Data Capturing

5.1 Student level- Individualized reports

Not only they get benchmarked scores, but they get advice on how to improve and development plan and advice.

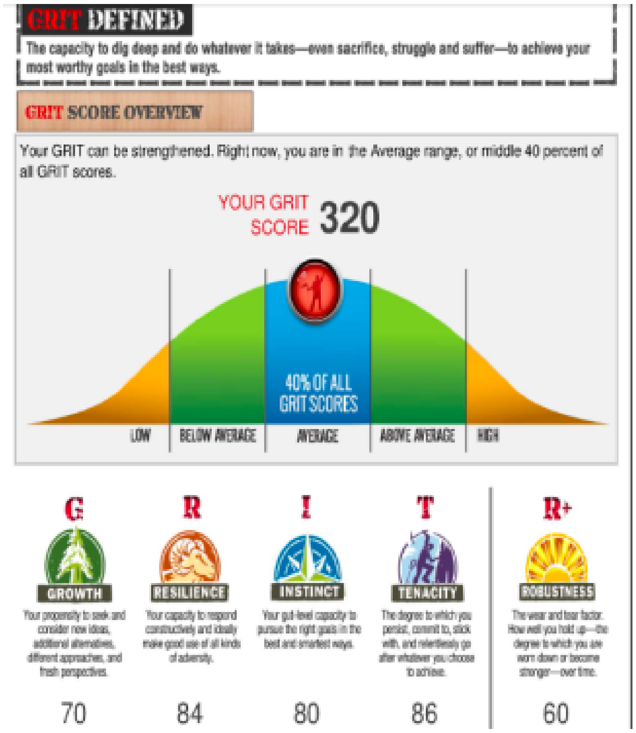

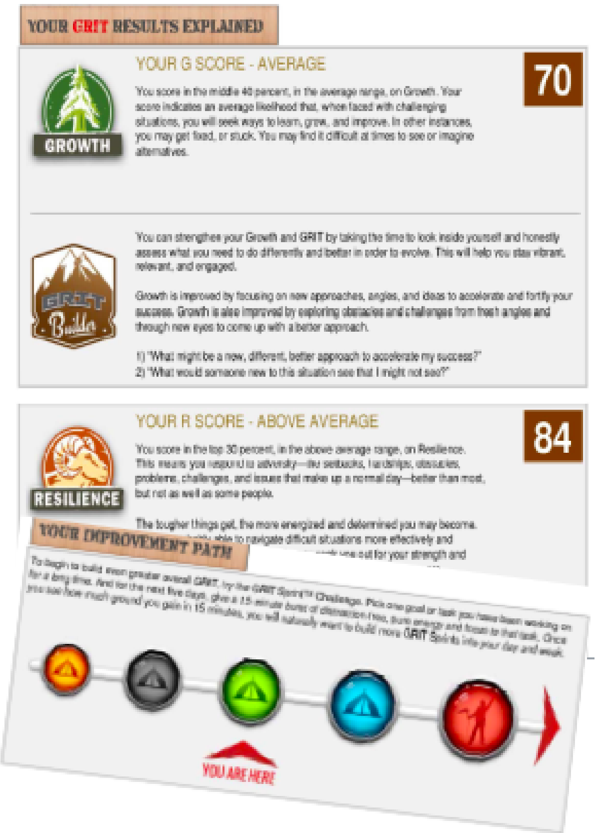

Pearson College and Career Readiness Solutions- All rights reserved® 2018

5.2 Student Advisor/ Career Coach Level- Measure student outcomes- Gradebook Capabilities

MyLab analyzes their results to provide personalized homework assignments focused on just the objectives they have not yet mastered. The gradebook allows you to track student performance as it corresponds to the learning outcomes for the course. Using Item Analysis, you're able to track class-wide understanding of particular objectives, so you can refine your program accordingly. With a few clicks, you can send personal feedback on a job well done or intervene for struggling students. Just-in-time teaching has never been easier.

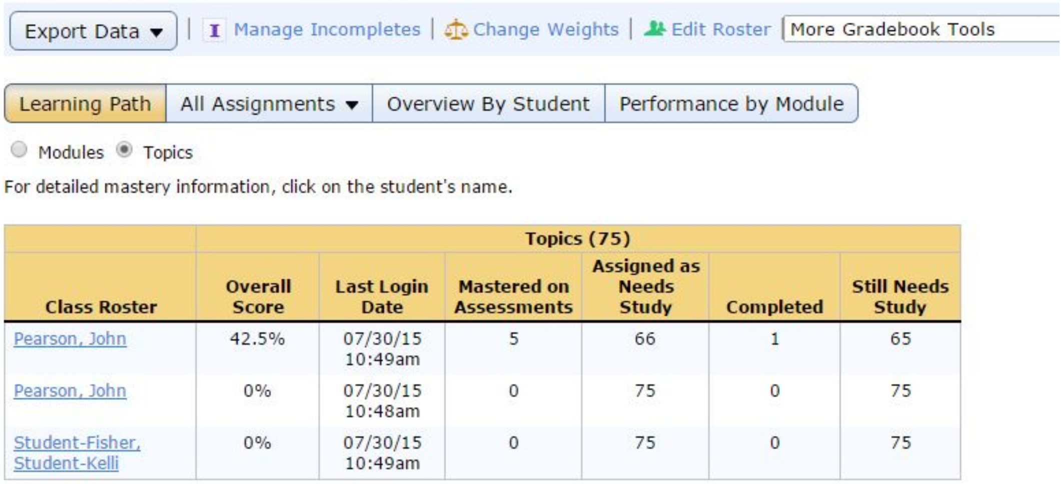

			Topics (75)			
Class Roster	Overall Score	Last Login Date	Mastered on Assessments	Assigned as Needs Study	Completed	Still Needs Study
Pearson, John	42.5%	07/30/15 10:49am	5	66	1	65
Pearson, John	0%	07/30/15 10:48am	0	75	0	75
Student-Fisher, Student-Kelli	0%	07/30/15 10:49am	0	75	0	75

5.3 Administrative and institutional Level- Advanced Reporting Dashboard

View, analyze, and report learning outcomes clearly and easily, MBRU can get the information they need to keep their students on track throughout the course, with the new Reporting Dashboard in MyLab from Pearson. Available via the Gradebook and fully mobile-ready,

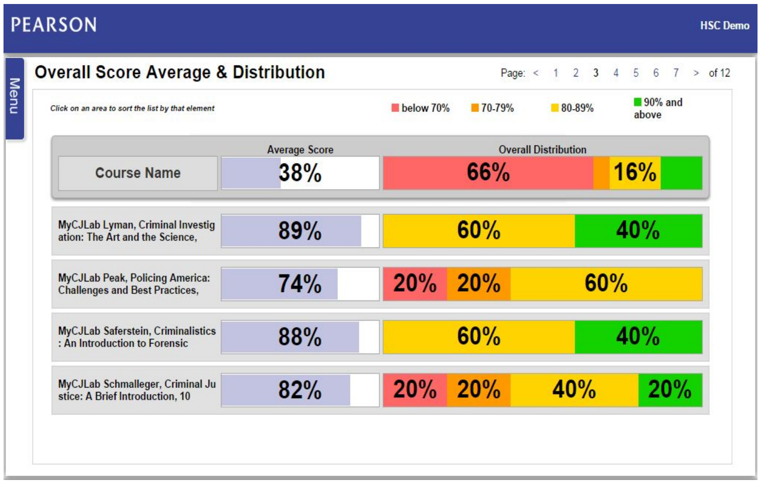

The Reporting Dashboard will allow educators to see what's working, as well as where they may need to change gears. Data on student performance by assignment, as well as student progression in the course overall, allows instructors to track students' understanding,

Pearson College and Career Readiness Solutions- All rights reserved® 2018

and intervene when necessary to lend a helping hand.

With just a few clicks, the Reporting Dashboard's fine-grain reports will allow administrators to compare performance across different courses, across individual sections of the same course, and within each course.

Five types of visual reports are included in this release:

- The Overall Score Average and Distribution report
- The Assignment Average Score and Time Spent report
- The Assignment Score Distribution report
- The Mastery report
- Item analysis

Item Analysis

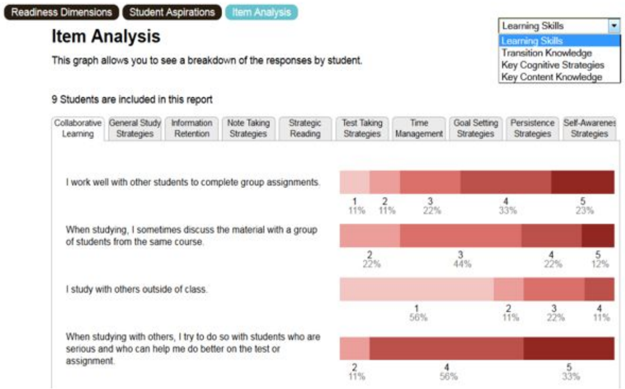

Readiness Dimensions

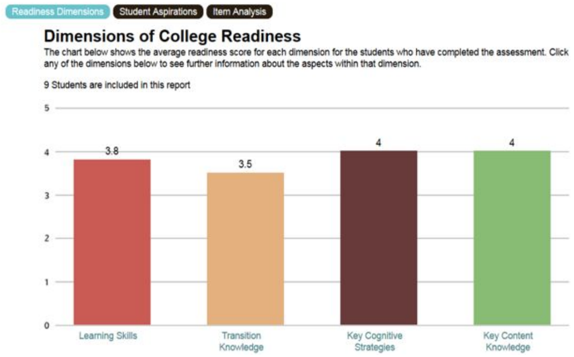

Student Aspirations

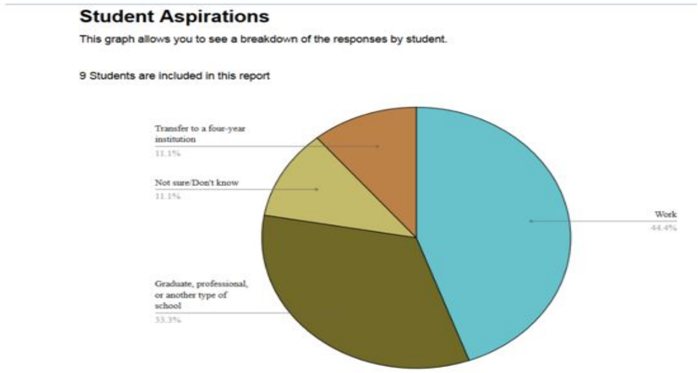

Pearson College and Career Readiness Solutions- All rights reserved® 2018
